# Systems-Level Phosphoproteomic and RPPA Profiling Reveals Stress and DNA Damage Signalling as Early Drivers of Polymyxin B Neurotoxicity

**DOI:** 10.1007/s12035-026-06126-x

**Published:** 2026-08-14

**Authors:** Maytham Hussein, Thuraya Safaa Ansaf, Terry C. C. Lim Kam Sian, Mark Baker, Pouya Faridi, Zhi Ying Kho, Nivedhitha Selvakumar, Keith S. Kaye, Gauri G. Rao, Jian Li, Tony Velkov

**Affiliations:** 1 Monash Biomedicine Discovery Institute, Department of Pharmacology, Monash University, Clayton, VIC 3800 Australia; 2 Clinical Proteomics Node, School of Clinical Sciences, Monash University, Clayton, VIC Australia; 3 Serere Medical Pty Ltd, 13 Featherway, Fletcher, NSW 2287 Australia; 4 Monash Biomedicine Discovery Institute, Department of Microbiology, Monash University, Clayton, VIC 3800 Australia; 5 Division of Allergy, Immunology and Infectious Diseases, Rutgers Robert Wood Johnson Medical School, New Brunswick, NJ USA; 6 Titus Family Department of Clinical Practice, USC Alfred E. Mann School of Pharmacy and Pharmaceutical Sciences, University of Southern California, 1985 Zonal Avenue, PSC 512 E, Los Angeles, CA 90089 USA; 7 School of Pharmacy, UNC Eshelman, University of North Carolina, Chapel Hill, NC USA

**Keywords:** Polymyxin B, Neurotoxicity, Central nervous system, Phosphoproteomics, Reverse phase protein array, Apoptosis, DNA damage signalling, Antibiotic toxicodynamics

## Abstract

Polymyxins remain indispensable last-line antibiotics for multidrug-resistant Gram-negative infections, yet their clinical use in central nervous system (CNS) infections is constrained by poorly understood neurotoxicity. Here, we define the early molecular signalling events underlying polymyxin B-induced CNS toxicity using an integrated phosphoproteomic and Reverse Phase Protein Array (RPPA) approach in rat brain following intracerebroventricular administration. Global phosphoproteomics revealed extensive phosphosite coverage but identified a highly selective set of significantly regulated phosphosites, implicating calcium-dependent signalling, transcriptional stress regulation, synaptic signalling, and cytoskeletal control, while parallel total proteomics showed minimal changes in protein abundance. RPPA profiling independently confirmed coordinated modulation of stress, apoptotic and survival-associated signalling pathways, including p53, CREB, SQSTM1, Bcl-2, and NFκB related nodes. Network and functional enrichment analyses converged on DNA damage signalling, apoptotic regulation and growth factor-mediated pathways as central features of the polymyxin B early neurotoxicity response, while phosphor to total protein analyses demonstrated suppression of proliferative and pro-survival signalling. Together, these data establish phosphorylation-driven signalling reprogramming as a primary early mechanism of polymyxin B-induced neurotoxicity, providing a mechanistic framework that links membrane-active antibiotic exposure to neuronal stress signalling and identifies candidate pathways for toxicity biomarkers and neuroprotective strategies.

**Supplementary Information:**

The online version contains supplementary material available at 10.1007/s12035-026-06126-x.

## Introduction

Polymyxins, (i.e., polymyxin B and colistin), have re-emerged as critical last-line antibiotics for the treatment of infections caused by multidrug-resistant (MDR) Gram-negative pathogens [1, 2]. Despite their clinical importance, polymyxins are associated with a narrow therapeutic window and dose-limiting toxicities, most notably nephron- and neuro-toxicity, which significantly constrain their safe clinical use [3–6]. While polymyxin-induced nephrotoxicity has been extensively studied, the molecular mechanisms underlying polymyxin-associated central nervous system (CNS) toxicity remain incompletely understood.

Clinical reports and experimental studies have documented a spectrum of neurotoxic manifestations following polymyxin exposure, including paresthesia, dizziness, confusion, seizures and neuromuscular blockade [4, 7, 8]. These effects are particularly relevant in clinical contexts where polymyxins gain access to the CNS, such as intraventricular or intrathecal administration for the treatment of MDR meningitis and ventriculitis [9–11]. Importantly, polymyxins neurotoxicity can occur in the absence of overt neuronal loss, suggesting that early functional and signalling perturbations may precede irreversible structural damage [12].

Emerging evidence indicates that polymyxin-induced neurotoxicity is mediated by complex molecular responses involving oxidative stress, mitochondrial dysfunction, dysregulated calcium signalling and activation of cellular stress pathways [12–15]. Our recent metabolomics-based investigations have demonstrated that polymyxin B induces rapid and profound metabolic reprogramming in the brain following intracerebroventricular (ICV) administration, implicating perturbations in energy metabolism, redox balance, and neurotransmitter-associated pathways as contributors to CNS neurotoxicity [14]. Complementary transcriptomic analyses further supported the activation of stress-responsive and damage-associated gene programs, highlighting a coordinated, multi-layered response to polymyxin B exposure [13]. Collectively, these findings suggested that rapid signalling-level regulation may represent an important upstream component of polymyxin B neurotoxicity and provided the rationale for the present phosphoproteomic and RPPA-based investigation.

Protein phosphorylation plays a central role in the rapid regulation of cellular signalling networks governing stress responses, apoptosis, DNA damage repair and neuronal survival [16]. Unlike changes in protein abundance, phosphorylation-dependent signalling events can occur within minutes to hours and are ideally suited to mediate early toxicodynamic responses that are important for understanding pharmacokinetics/pharmacodynamics/toxicodynamics (PK/PD/TD) relationships [17]. However, phosphorylation-level regulation in polymyxin-induced CNS toxicity has not been systematically investigated. RPPA technology provides a sensitive, high-throughput platform for quantifying both total and phospho-specific protein states across multiple signalling pathways, enabling robust interrogation of phosphorylation-dependent signalling alterations in complex neuronal tissues such as the brain and spinal cord [18].

In this study, we integrated global phosphoproteomic profiling with targeted RPPA analysis to characterize phosphorylation-dependent signalling responses in rat brain following intracerebroventricular administration of clinically relevant concentrations of polymyxin B. In order to delineate the signalling protein-level architecture underlying early polymyxin B-induced neurotoxic responses, we employed unbiased phosphosite-level discovery with pathway-focused protein and phosphoprotein quantification, together with unsupervised multivariate analyses, differential phosphorylation testing, protein–protein interaction network analysis and Gene Ontology enrichment. Our results demonstrate selective remodelling of stress, apoptosis and DNA damage-associated signalling pathways at the phosphorylation levels, occurring in the absence of widespread changes in total protein abundance. These findings establish phosphorylation-driven signalling reprogramming as a central early mechanism of polymyxin-associated CNS toxicity.

## Results

### Phosphorylation-Level Neuronal Remodelling Following Polymyxin B Exposure

**Fig. 1:**
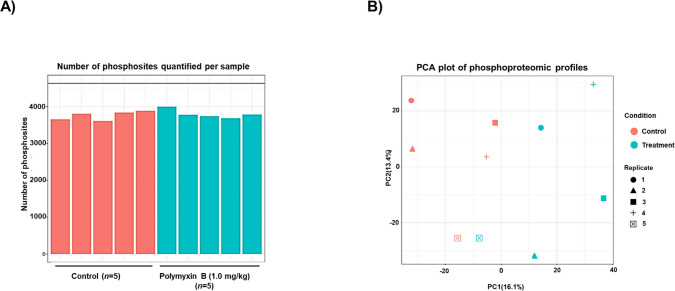
Global phosphoproteomic landscape and treatment-associated signalling differences in rat brain tissue following polymyxin B exposure.** A** Number of phosphosites quantified per sample in control and polymyxin B-treated rat brain samples. **B** Principal component analysis (PCA) of phosphoproteomic profiles showing sample distribution based on quantified phosphosites

Global phosphoproteomic profiling was performed using ZrIMAC-based phosphopeptide enrichment coupled with FAIMS-assisted LC–MS/MS acquisition to interrogate phosphorylation changes in rat brain following polymyxin B exposure (1.0 mg/kg). Across all samples, approximately 4,000 phosphosites consistently quantified at a 1% false discovery rate. Phosphopeptide enrichment and quantification were reproducible across biological replicates, with comparable phosphosites coverage per sample (Fig. 1A).

Unsupervised analyses demonstrated treatment-associated differences at the phosphorylation level. Principal component analysis showed separation between untreated control and polymyxin B-treated groups, indicating distinct phosphosignaling states (Fig. 1B). Consistently, hierarchical clustering of differentially regulated phosphosites revealed condition-dependent phosphorylation patterns across samples (Figure S1). Variability within each condition was within the expected range for phospho-enriched datasets, supporting data robustness (Figure S2).

Applying stringent statistical thresholds (log₂ fold change ≥ 1, adjusted *p* < 0.05), 14 phosphosites were identified as significantly regulated following polymyxin B exposure (Table 1). These sites mapped to proteins involved in calcium/calmodulin-dependent signalling (CAMK2A, CAMK2G), transcriptional stress regulation (FOXO1), synaptic/membrane-associated signalling (ADCY5, GRM1, SH3GL2) and cytoskeletal regulation (PHACTR1, DLGAP4). Both increases and decreases in phosphorylation were observed, indicating selective modulation of discrete signalling nodes rather than widespread phosphorylation changes.

**Table 1:** Significantly regulated phosphosites (log₂ fold change ≥ 1, adjusted *p* < 0.05) following polymyxin B exposure

Phospho-peptide sequence	Gene	Protein name	Phosphosite(s)	log₂ fold change	Adjusted *p* value
SELALAPAPAPAATHSPLHR	Sphk2	Sphingosine kinase 2	S379	−2.96	0.0059
QLVYEADGCSPHGTLK	IQSEC2	IQ motif and Sec7 domain ArfGEF 2	S627	−3.28	0.00937
EGTQASEGYFSQSQEEEFAQSEEPCAK	Dbn1	Drebrin	S658	3.62	0.036
SVSPPGYAAQTAASPAPR	ADCY5	Adenylate cyclase type 5	S8	2.46	0.0059
MLTINPSKR	CAMK2A	Calcium/calmodulin-dependent protein kinase type II subunit alpha	T253	−4.06	0.000378
GAFSVVR	CAMK2G	Calcium/calmodulin-dependent protein kinase type II subunit gamma	S25	−2.65	0.00675
HLSDSLDPPHEPLFAGPDR	DLGAP4	Disks large-associated protein 4	S13	−2.7	0.0147
ASNGDTPTHEDLTK	EPB4.1	Protein 4.1	T60	3.16	0.0121
LSPIMTEQDDLGDGDVHSLVYPPSAAK	FOXO1	Forkhead box protein O1	S323	2.74	0.00153
LTPEDSPALTPPSPFR	GRM1	Metabotropic glutamate receptor 1	T1151	2.54	0.0147
SDSLVPGTHTPPIR	PHACTR1	Phosphatase and actin regulator 1	S97	−2.73	0.0121
MSLEFATGDGTQPNGGLSHTGTPK	SH3GL2	Endophilin-A1	S262	3.46	0.00675
EKPPYGKSPVVMVDEILSSSPPK	SLC24A4	Slc24a4 protein	S249	−3.98	0.0018
LDGSHQELETLK	ZWINT	ZW10 interactor	S209	−1.9	0.0059

In contrast, parallel total proteomics analysis identified approximately 6,100 proteins, of which ~ 4,100 were quantifiable; however, only three proteins met criteria for significant differential abundance (Figure S3A-B). This limited abundance-level response indicates that early rapid-onset polymyxin B-induced effects are primarily mediated through more dynamic phosphorylation-dependent signalling rather than changes in protein expression, which may be delayed and occurred over a longer timeframe.

### RPPA-Based Proteomic Profiling Reveals Stress- and Apoptosis-Associated Signalling Responses to Polymyxin B Exposure

To complement the global phosphoproteomic analysis and to assess pathway-level signalling alterations at the protein and phosphoprotein level, RPPA profiling was performed on rat brain samples following intracerebroventricular polymyxin B exposure. A curated panel of 75 total and phosphorylation-specific antibodies targeting key signalling pathways was analyzed.

**Fig. 2:**
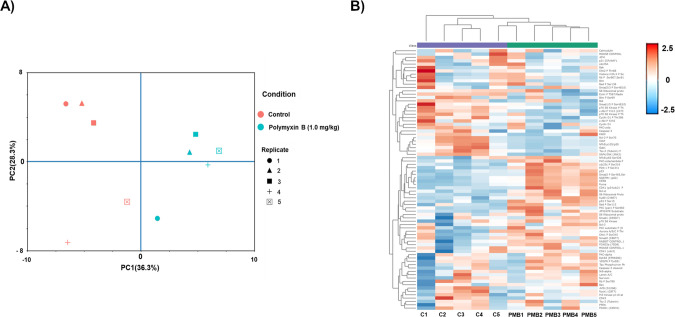
RPPA-based proteomic profiling reveals treatment-associated signalling alterations following intracerebroventricular polymyxin B exposure. **A** Principal component analysis (PCA) of RPPA data derived from total and phospho-protein measurements. **B** Hierarchical clustering heatmap of RPPA targets across control and polymyxin B-treated samples. Rats received intracerebroventricular polymyxin B (1 mg/kg), and brain tissue was collected 1 h post-injection (*n* = 5 biological replicates per group). C = Control, PMB = polymyxin B

Unsupervised multivariate analysis of RPPA profiles demonstrated clear treatment-associated differences. Principal component analysis revealed separation between control and polymyxin B-treated samples along the first principal component, indicating coordinated alterations in protein signalling states following treatment (Fig. 2A). Hierarchical clustering further supported this distinction, with samples grouping according to treatment condition and displaying consistent within-group RPPA signatures (Fig. 2B).

**Fig. 3:**
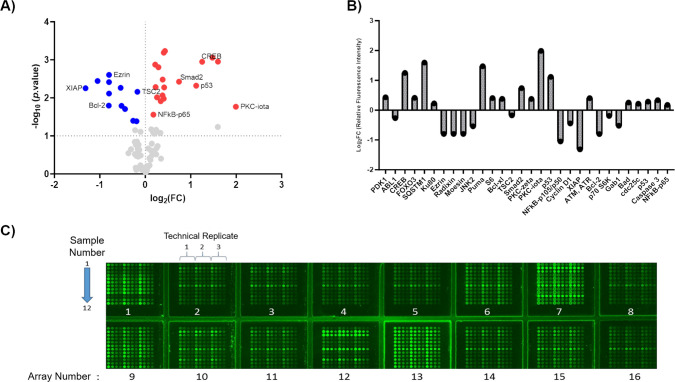
Differential RPPA responses to intracerebroventricular polymyxin B exposure.** A** Volcano plot displaying log₂ fold change *vs.* log₁₀(*p*) value for RPPA targets comparing polymyxin B-treated and control samples. **B** Bar plot showing log₂ fold changes for selected RPPA targets. **C** Complete FastGreen stained RPPA slide showing the printing layout used for all reverse-phase protein arrays. Biological samples were printed in sample positions 1–12 (positions 5 and 6 unused), with each sample printed as three technical replicates comprising a four-point dilution series (1.5, 0.75, 0.375 and 0.1875 mg/mL). Subarrays are numbered 1–16. The same printing layout was used for all antibody-probed RPPA slides. Rats received intracerebroventricular polymyxin B (1 mg/kg), and brain tissue was collected 1 h post-injection (*n* = 5 biological replicates per group)

Differential expression analysis identified a subset of RPPA targets exhibiting notable changes in response to polymyxin B exposure. Volcano plot analysis illustrated the overall distribution of effect sizes and statistical significance, highlighting a limited number of proteins with pronounced changes relative to control (Fig. 3A). These alterations predominantly reflected focused modulation of stress response, apoptotic, survival and cytoskeletal signalling pathways rather than widespread proteomic remodelling.

To illustrate the direction and magnitude of these changes, log₂ fold changes (i.e., polymyxin B *vs.* untreated control) for selected RPPA targets are summarized in Fig. 3B. Increased RPPA signal intensities were observed for proteins associated with cellular stress and damage responses, including SQSTM1 (p62), CREB, Puma and one p53 antibody; consistent with activation of stress-responsive and pro-apoptotic signalling pathways. In contrast, reduced signal intensities were observed for proteins involved in cell survival and cytoskeletal organization, including XIAP, Bcl-2, NFκB-p105/p50 and members of the Ezrin/Radixin/Moesin complex, suggesting attenuation of survival signalling and structural regulatory pathways. Figure 3C shows the complete FastGreen stained RPPA printing layout used for all arrays. The mapping between slide number, array number and antibody identity is provided in Supplementary Table S1, and the complete RPPA dataset is available in Supplementary Data S1.

**Fig. 4:**
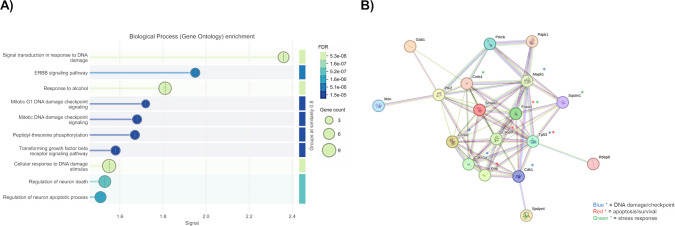
Functional enrichment and protein–protein interaction analysis of RPPA targets.** A** Gene Ontology (GO) Biological Process enrichment analysis of differentially responsive RPPA targets following intracerebroventricular (ICV) polymyxin B treatment in rat brain. Enriched terms are displayed with corresponding signal strength, false discovery rate (FDR), and gene counts. **B** Protein–protein interaction (PPI) network of the same RPPA targets generated using STRING, illustrating interaction relationships among proteins included in the enrichment analysis

Functional interpretation of the differentially responsive RPPA targets was supported by Gene Ontology enrichment and protein–protein interaction analyses. Gene Ontology analysis highlighted biological processes related to DNA damage signalling, apoptotic regulation and growth factor-mediated signalling pathways (Fig. 4A). Consistently, protein–protein interaction network analysis revealed a tightly connected signalling module centred on stress response, apoptosis and DNA damage-associated proteins (Fig. 4B).

**Fig. 5:**
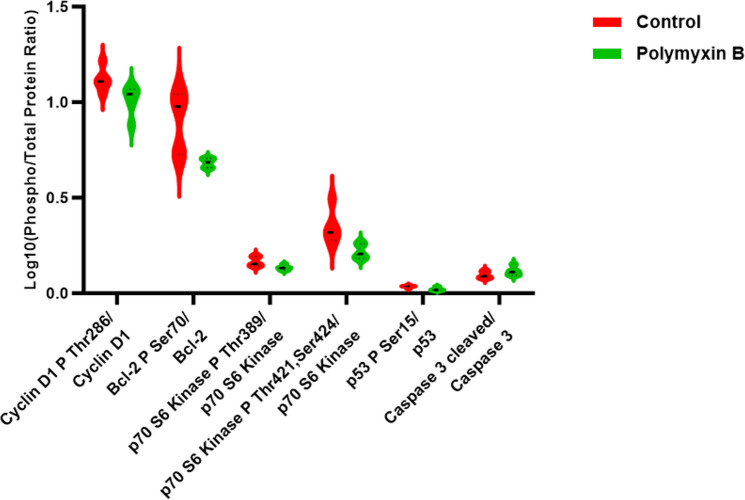
Phospho-to-total protein activation ratios following intracerebroventricular polymyxin B exposure. Log₂-transformed phospho-to-total protein activation ratios for selected signalling proteins in rat brains following intracerebroventricular (ICV) polymyxin B treatment. Violin plots show the distribution of normalized phosphorylation ratios in control (red) and polymyxin B-treated (green) groups (*n* = 5 biological replicates per group). Statistical significance was assessed using a two-tailed unpaired t-test; significance thresholds are indicated as *p* < 0.05

Finally, phosphorylation-specific RPPA measurements were normalized to corresponding total protein levels to assess changes in protein activation states. Analysis of phospho-to-total protein ratios demonstrated selective reductions in activation for Cyclin D1, Bcl-2, and p70 S6 kinase phosphorylation sites following polymyxin B exposure, consistent with dysregulation of proliferative and survival-associated signalling pathways (Fig. 5). Together, these RPPA findings indicate that polymyxin B induces targeted alterations in stress, apoptosis and survival-related signalling networks at the protein level, complementing the phosphoproteomic data and supporting a signalling-driven mechanism underlying early onset polymyxin B-induced neurotoxicity.

## Discussion

Polymyxins are retained as critical last-line therapeutic options against MDR Gram-negative infections, particularly CNS infections where intrathecal or intracerebroventricular administration is sometimes required [12, 19]. However, polymyxin-associated neurotoxicity, including seizures, altered consciousness, neuromuscular blockade and encephalopathy, has been consistently reported in both clinical and preclinical studies; limiting the therapeutic window [4, 15, 20, 21]. Despite decades of clinical use, the molecular signalling events underlying polymyxin-induced CNS toxicity have remained poorly defined.

In the present study, RPPA-based proteomic profiling revealed that clinically relevant polymyxin B exposure via intracerebroventricular administration induces coordinated remodelling of stress, apoptosis and DNA damage associated signalling pathways in rat brain tissue. Protein–protein interaction network analysis identified a densely interconnected signalling module centred on canonical regulators of cellular stress and survival, including MAPK, AKT, p53-associated nodes and apoptosis-related proteins. This tightly coupled network structure indicates that early onset polymyxin B-induced neurotoxicity is not driven by isolated protein perturbations but instead reflects system-level signalling reprogramming.

These findings align closely with our prior mechanistic studies demonstrating that polymyxins disrupt neuronal membrane integrity, perturb calcium homeostasis and induce mitochondrial dysfunction [12, 14]. Mitochondrial injury and oxidative stress are well-recognized upstream triggers of DNA damage response signalling and apoptotic pathway activation, providing a mechanistic link between polymyxin membrane activity and the intracellular signalling changes observed here. Indeed, our transcriptomic analyses of intracerebroventricular polymyxin B administration have reported early induction of stress-response genes, inflammatory mediators, and pathways associated with neuronal injury and repair, preceding overt histopathological damage [13].

Several significantly regulated phosphosites identified in the present study were linked to neuronal stress and calcium-dependent signalling pathways. Reduced phosphorylation of CAMK2-associated sites suggests disruption of calcium/calmodulin-regulated neuronal signalling, consistent with previous reports of altered calcium homeostasis during polymyxin-associated neurotoxicity [12, 14]. Increased FOXO1 phosphorylation is indicative of stress-responsive transcriptional regulation associated with oxidative stress and apoptotic signalling. In addition, altered phosphorylation of GRM1, ADCY5 and SH3GL2 further points toward perturbation of synaptic and membrane-associated signalling processes. These signalling alterations are consistent with rapid intracellular stress responses occurring following polymyxin B exposure.

Gene Ontology enrichment analysis further supports this interpretation, revealing significant enrichment of biological processes related to DNA damage signalling, mitotic checkpoint regulation, protein phosphorylation and apoptotic control. The prominence of DNA damage-associated pathways is particularly noteworthy, as polymyxins are not classically considered genotoxic agents. Instead, emerging evidence suggests that secondary oxidative stress, metabolic disruption and calcium dysregulation can activate DNA damage signalling cascades without direct DNA lesions [12, 22, 23]. This is consistent with metabolomics studies demonstrating polymyxin B induced perturbations in brain energy metabolism, redox balance and neurotransmitter pathways following CNS exposure [14].

The enrichment of growth factor-related signalling pathways, including ERBB and TGF-β receptor signalling, suggests an attempted compensatory or survival response within neural tissue [24, 25]. Growth factor signalling plays a central role in neuronal maintenance, synaptic plasticity, recovery following injury and dysregulation of these pathways has been implicated in neurotoxic and neurodegenerative processes [26, 27]. Disruption of these protective signalling axes by polymyxin B exposure may, therefore, contribute to impaired neuronal resilience and delayed functional recovery.

Importantly, the RPPA data indicate that these signalling alterations occur in the absence of widespread changes in total protein abundance, supporting the conclusion that early polymyxin B neurotoxicity is driven predominantly by dynamic post-translational signalling events rather than transcriptional or translational reprogramming. This paradigm is consistent with broader phosphoproteomic and metabolomic literature demonstrating that cellular stress responses, particularly following antibiotic exposure, are initially mediated by rapid signalling and metabolic adaptations that precede detectable changes in protein abundance. In the context of polymyxin B exposure, this is further supported by our previous metabolomics-based analyses showing early metabolic and redox perturbations in the brain following intracerebroventricular polymyxin B administration [14].

**Fig. 6:**
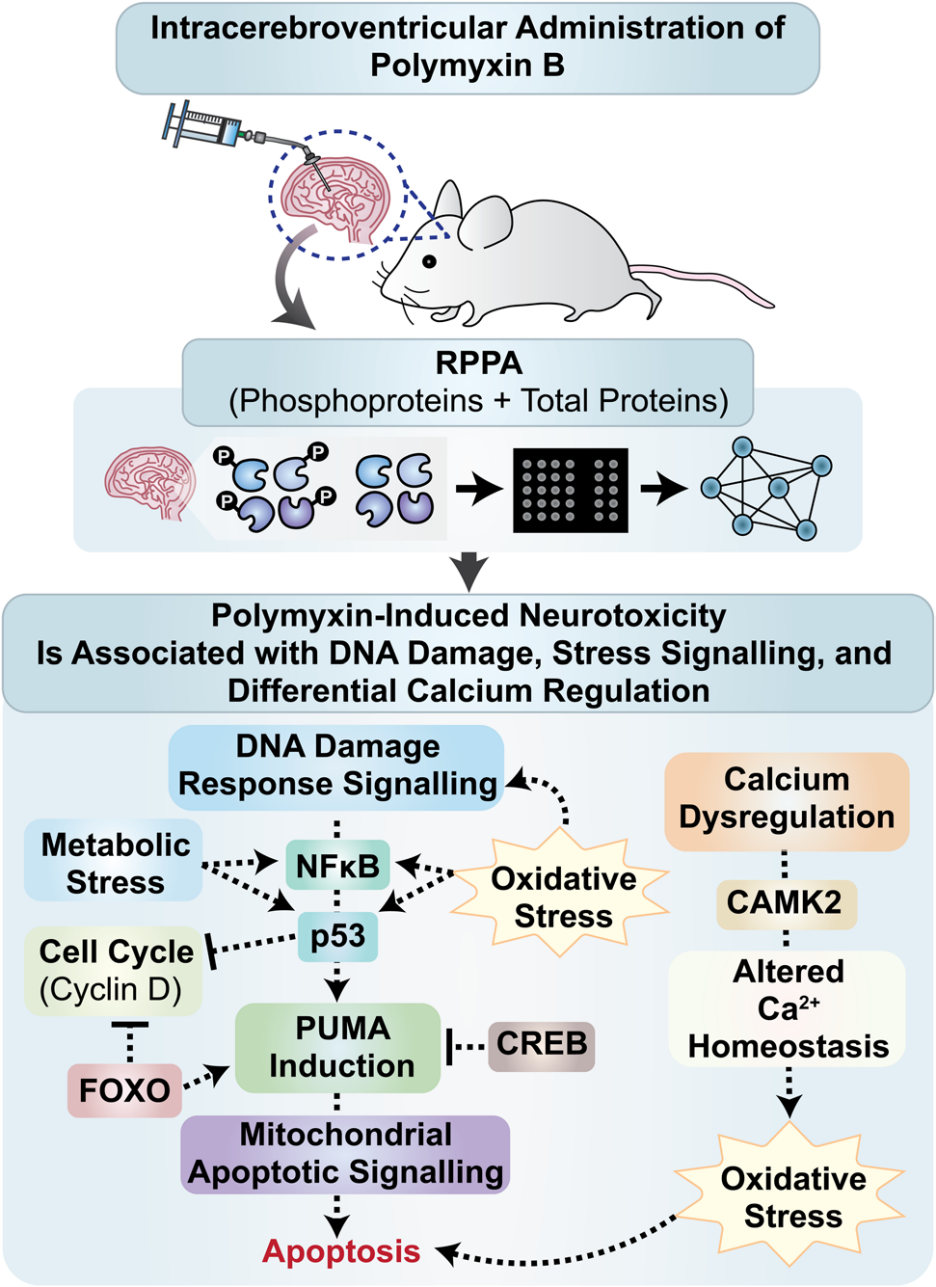
Integrated mechanistic framework of early polymyxin B-induced CNS neurotoxicity. Schematic model summarizing signalling alterations identified by global phosphoproteomics and RPPA profiling following intracerebroventricular (ICV) polymyxin B (1.0 mg/kg) exposure in rat brain. Polymyxin B exposure is associated with metabolic stress, oxidative stress, and altered calcium homeostasis (CAMK2-related signalling), converging on activation of DNA damage response signalling, transcriptional stress regulators (NFκB, CREB, FOXO), cell-cycle modulation (Cyclin D), and p53-dependent induction of PUMA. These coordinated signalling alterations are linked to mitochondrial apoptotic signalling and apoptosis

These findings support a mechanistic framework in which polymyxin B exposure is associated with early membrane and mitochondrial stress, accompanied by amplification of intracellular stress signalling, activation of DNA damage response pathways, and engagement of apoptotic regulatory networks (Fig. 6). This integrated signalling architecture provides a plausible molecular explanation for the early neurological adverse effects observed following CNS polymyxin B administration.

This study provides the first RPPA-based and phosphoproteomics systems-level characterization of polymyxin B induced signalling alterations in the brain. By integrating proteomic profiling with protein–protein interaction and Gene Ontology analyses, we demonstrate that polymyxin exposure triggers coordinated activation of stress response, DNA damage-associated, and apoptotic signalling pathways rather than isolated molecular disruptions. These findings extend existing transcriptomic and metabolomic evidence and establish a mechanistic bridge between polymyxin membrane activity and downstream neuronal injury signalling [13, 14].

Understanding polymyxin B neurotoxicity as an active, regulated biological process rather than non-specific toxicity has important translational implications. The signalling nodes identified here may serve as early biomarkers of CNS toxicity and suggest potential targets for neuroprotective intervention. Ultimately, integrating such mechanistic insights with optimized dosing strategies and PK/PD/TD modelling will be essential to improve the safety of polymyxin B therapy for life-threatening CNS infections.

## Materials and Methods

### Ethics Approval

All animal experiments were approved by the Animal Ethics Committee of the University of Melbourne (IACUC #1,914,890). All procedures were conducted in accordance with the Australian Code for the Care and Use of Animals for Scientific Purposes.

### Reagents

Polymyxin B sulfate was obtained from Beta Pharma (China; batch #20,120,204). Drug solutions were freshly prepared in sterile 0.9% sodium chloride and vortexed until fully dissolved immediately prior to administration. All reagents used for protein extraction and downstream analyses were of analytical or LC–MS grade.

### Animal Experimental Design and Intracerebroventricular Administration

Adult male Sprague–Dawley rats were housed under controlled environmental conditions (21 °C, 12-h light/dark cycle) with ad libitum access to food and water. Animals were group-housed prior to surgery and singly housed following cannulation to prevent disruption of cranial mounts.

Stereotaxic implantation of a guide cannula into the right lateral ventricle was performed under general anaesthesia. Following a 24-h recovery period, animals received a single intracerebroventricular administration of polymyxin B (1.0 mg/kg) or vehicle control (0.9% NaCl). Drug delivery was performed using an internal cannula connected to a Hamilton microsyringe via polyethylene tubing. Injection volumes were adjusted according to body weight (approximately 4–6 µL). The internal cannula was maintained in position for 10 s following injection to minimize reflux.

### Intracerebroventricular Drug Administration

Polymyxin B (1.0 mg/kg) or an equivalent volume of 0.9% sodium chloride was administered via the implanted guide cannula using an internal cannula connected to a Hamilton micro-syringe (Hamilton, NV, USA) via PE-50 tubing [28]. Injection volumes (approximately 4–6 µL) were calculated based on body weight. The internal cannula was maintained in position for 10 s post-injection to prevent backflow.

### Tissue Collection and Sample Preparation

At 1 h following intracerebroventricular administration, animals were deeply anesthetized using isoflurane and euthanized by cardiac puncture. Brains were rapidly removed, and regions of interest were dissected on an ice-cold matrix. Brain tissues were snap-frozen on dry ice and stored at −80 °C until further processing.

For RPPA analysis, frozen brain tissues were homogenized in lysis buffer containing protease and phosphatase inhibitors. Lysates were clarified by centrifugation, and total protein concentrations were determined prior to array printing.

### Global Phosphoproteomics Analysis

#### Protein Extraction and Phosphopeptide Enrichment

SDC lysis buffer (4% sodium deoxycholate, 100 mM Tris–HCl pH 8.5) and immediately heat-treated at 95 °C for 5 min. Phosphoproteomics enrichment was performed as previously described [29]. In brief, the lysates were homogenized by sonication, and a 350µg aliquot was taken post BCA assay. Disulfide bonds and carbamidomethylated cysteine residues were reduced using Reduction/Alkylation Buffer (100 mM TCEP and 400 mM 2-chloroacetamide pH 7) and heated at 95 °C for 5 min. The samples were allowed to cool down and were then subjected to Trypsin and LysC digestion (2 µg of each enzyme per 200 µg of protein) overnight at 37 °C with shaking (1500 RPM). For the total proteomics analysis, 50ug of the overnight digest was cleaned using SDB-RPS (Styrenedivinylbenzene–Reversed Phase Sulfonate) stage tips.

The remainder of the lysates were subjected to phosphoproteomiocs enrichment on the Thermo Kingfisher platform using MagReSyn® Zr-IMAC HP magnetic microbeads (Resyn Bioscinces). C18 desalted peptides were completely dried down and resuspended in 200 µL Equilibration Buffer (80% acetonitrile (ACN), 5% trifluoric acid (TFA) and 1M glycolic acid). The peptides were then transferred onto the Kingfisher, prior to being subjected to enrichment onto the ZrIMAC microbeads, followed by a series of washes with the Equilibration Buffer before elution with 1% NH3OH/50% ACN. The eluates were dried down to completeness and subjected to SDB-RPS clean-up.

The SDB-RPS clean-up eluate of both the proteomics and phosphoprotomics were completely dried using a vacuum concentrator. The peptides were reconstituted in the loading buffer (0.1% FA/2% ACN) prior to mass spectrometry analysis.

#### LC–MS/MS Acquisition

For the mass spectrometry analysis, the samples were analysed on an UltiMate 3000 RSLC nano LC system (Thermo Fisher Scientific) coupled to a Thermo Orbitrap 480 mass spectrometer (Thermo Fisher Scientific). Peptides were loaded via an Acclaim PepMap 100 trap column (100 μm × 2 cm, nanoViper, C18, 5 μm, 100 Å, Thermo Fisher Scientific) and subsequent peptide separation was on an Acclaim PepMap RSLC analytical column (75 μm × 50 cm, nanoViper, C18, 2 μm, 100 Å, Thermo Fisher Scientific). For each liquid chromatography-tandem mass spectrometry (LC–MS/MS) analysis, 1 μg of peptides as measured by a nanodrop 1000 spectrophotometer (Thermo Fisher Scientific) was loaded on the pre-column with microliter pickup. Peptides were eluted using a 2 h linear gradient of 80% ACN/0.1% FA at a flow rate of 250 nL/min using a mobile phase gradient of 2.5–42.5% ACN. The mass spectrometry data was acquired in data-dependent acquisition (DDA) mode as previously described (Lim Kam Sian et al. 2024). During the acquisition on the Orbitrap Exploris 480 instrument, full scan MS (m/z 350–1200) was performed in the Orbitrap at two different Field Asymmetric Waveform Ion Mobility Spectrometry (FAIMS) collision voltage (CV) of 45 and 65. The resolution was set to 60,000 after accumulating ions to a normalized AGC target of 300% with a custom maximum injection time set to Auto. Dynamic exclusion was applied, and MS2 selection was carried out with a fixed cycle time of 1.5 s and an intensity threshold of 5 × 104 and including charge states 2–7. The Orbitrap MS2 scans had an isolation window of 1.4 m/z, HCD collision energy of 28%, resolution of 30,000, a custom AGC target with a normalized value of 100%, and a custom maximum injection time set to Auto.

#### Data Processing and Quantification

Raw mass spectrometry data were processed using MaxQuant (v2.0.3.1) with the Andromeda search engine. Searches were performed against a reviewed protein database with a 1% false discovery rate applied at both peptide and protein levels. Carbamidomethylation of cysteine was specified as a fixed modification, while oxidation (M), *N*-terminal acetylation and phosphorylation of serine, threonine and tyrosine were included as variable modifications.

Label-free quantification was applied to phosphosites. Across all samples, approximately 11,000 unique phosphosites mapping to ~ 3,400 proteins were identified, with ~ 4,000 phosphosites consistently quantified. Differential phosphorylation was assessed using log₂ fold change thresholds ≥ 1 and adjusted *p* values < 0.05. Unsupervised analyses, including principal component analysis and hierarchical clustering, were used to evaluate global phosphoproteomic structure and condition-specific signalling patterns. Processed total proteomics and phosphoproteomics datasets used for downstream statistical analyses are provided in Supplementary Tables S2 and S3, respectively.

### Reverse Phase Protein Array (RPPA) Analysis

#### RPPA Platform and Slide Processing

RPPA analysis was performed using a validated, high-throughput nitrocellulose-based RPPA platform following established core facility standard operating procedures. Brain protein lysates were serially diluted and printed in technical triplicates onto nitrocellulose-coated slides. Each slide was probed with a single, highly validated antibody targeting either total or phospho-specific protein epitopes. A complete list of antibodies used for RPPA analysis, including phosphosite-specific epitopes, is provided in Supplementary Table S4.

#### Signal Detection and Normalization

Slides were stained with FastGreen to quantify total protein content and scanned under multiple photomultiplier tube (PMT) settings to ensure signal detection within the linear dynamic range. Raw fluorescence signals were background-subtracted and normalized to total protein levels. Quality control metrics included assessment of technical replicate variability, dilution linearity, and dynamic range. For each antibody, the optimal PMT setting was selected based on linearity and signal-to-noise performance. The complete FastGreen RPPA printing layout is shown in Fig. 3C. The complete mapping between slide number, array number and antibody identity is provided in Supplementary Table S1, and the complete RPPA dataset is supplied as Supplementary Data File S1.

### Data Analysis

Normalized RPPA data were log₂-transformed prior to statistical analysis. Differential protein abundance and phosphorylation changes between control and polymyxin B treated groups were assessed using two-tailed unpaired t-tests, with significance thresholds defined as *p* < 0.05 unless otherwise specified. Multivariate analyses, including principal component analysis and hierarchical clustering, were used to assess global proteomic structure and treatment-associated patterns.

### Functional Enrichment and Network Analysis

Proteins exhibiting significant changes in RPPA analyses were subjected to protein–protein interaction (PPI) network analysis using STRING. Gene Ontology (GO) Biological Process enrichment analysis was performed to identify overrepresented functional pathways. Network visualization and enrichment analyses were used to contextualize altered signalling pathways associated with polymyxin B exposure.

### Data Visualization

Figures including phosphosite quantification, PCA plots, hierarchical clustering heatmaps, volcano plots, bar graphs, and violin plots were generated using GraphPad Prism and R-based visualization tools. All plots were derived from normalized datasets as described above.

## Supplementary Information

Below is the link to the electronic supplementary material.

ESM1 (DOCX 240 KB)

## Data Availability

The mass spectrometry phosphoproteomics data have been deposited in the ProteomeXchange Consortium via the PRIDE repository under accession number PXD075314. Reviewer access details Log in to the PRIDE website using the following details: Project accession: PXD075314 Token: wzRx444fLBrZ Alternatively, reviewer can access the dataset by logging in to the PRIDE website using the following account details: Username: reviewer\_pxd075314@ebi.ac.uk Password: vuKUsXwfBSYl Raw RPPA TIFF slide images (antibody-stained and FastGreen total protein scans) are available from the corresponding authors upon request.
