# Supplementary material for: Systems-Level Phosphoproteomic and RPPA Profiling Reveals Stress and DNA Damage Signalling as Early Drivers of Polymyxin B Neurotoxicity
Source: Mol Neurobiol. 2026 Aug 14;63(1):835. doi: 10.1007/s12035-026-06126-x (PMC13476288; doi:10.1007/s12035-026-06126-x)
Supplement: Supplementary file 1 [file 12035_2026_6126_MOESM1_ESM.docx]

**Systems-level phosphoproteomic and RPPA profiling reveals stress and DNA damage signalling as early drivers of polymyxin B neurotoxicity**

Maytham Hussein,^1^* Thuraya Safaa Ansaf,^1^ Terry C. C. Lim Kam Sian,^2^ Mark Baker,^3^ Pouya Faridi,^2^ Zhi Ying Kho,^4^ Nivedhitha Selvakumar,^2^ Keith S Kaye,^5^ Gauri G. Rao,^6,7^ Jian Li,^4^ Tony Velkov^1^*

**Affiliations:**^1^Monash Biomedicine Discovery Institute, Department of Pharmacology, Monash University, Clayton, Victoria, 3800, Australia; ^2^Clinical Proteomics Node, School of Clinical Sciences, Monash University, Clayton, VIC, Australia;^3^ Serere Medical Pty Ltd 13 Featherway, Fletcher, NSW 2287, Australia; ^4^Monash Biomedicine Discovery Institute, Department of Microbiology, Monash University, Clayton, Victoria, 3800, Australia; ^5^Division of Allergy, Immunology and Infectious Diseases, Rutgers Robert Wood Johnson Medical School, New Brunswick, NJ, USA; ^6^Titus Family Department of Clinical Practice, USC Alfred E. Mann School of Pharmacy and Pharmaceutical Sciences, University of Southern California, 1985 Zonal Avenue, PSC 512 E, Los Angeles, CA 90089, USA; ^7^ UNC Eshelman School of Pharmacy, University of North Carolina, Chapel Hill, North Carolina, USA

*Corresponding authors: tony.velkov@monash.edu; maytham.hussein.old@monash.edu

**Keywords.** Polymyxin B; neurotoxicity; central nervous system; phosphoproteomics; reverse phase protein array; apoptosis; DNA damage signalling; antibiotic toxicodynamics.

**Short Title:** Early Signalling Events in Polymyxin B CNS Toxicity

**Figure S1.** Hierarchical clustering heatmap of phosphosite intensities across control and polymyxin B-treated samples. Rats received intracerebroventricular polymyxin B (1 mg/kg), and brain tissue was collected 1 h post-injection (*n* = 5 biological replicates per group).

**Figure S2. Dataset variability in phosphoproteomic analysis.** Coefficient of variation distributions for quantified phosphosites across control and polymyxin B treated samples indicate comparable variability between conditions and fall within the expected range (~30-50%) for phospho-enriched datasets.

**Supplementary Figure S3.** Global total proteomics profiling following intracerebroventricular polymyxin B exposure. Rats received intracerebroventricular polymyxin B (1 mg/kg), and brain tissue was collected 1 h post-injection (*n* = 5 biological replicates per group). **(A)** Principal component analysis (PCA) of total proteomic profiles showing comparatively modest abundance-level separation between control and polymyxin B-treated samples. **(B)** Hierarchical clustering heatmap of significantly altered proteins identified in the total proteomics dataset. Rows represent proteins and columns represent biological replicates. Colour scale indicates relative protein abundance following row-wise normalization.

**Supplementary Table S1.** RPPA antibody array map. Slide number, array number, array location, antibody identity and antibody type for all reverse-phase protein array (RPPA) slides used in this study.

| Slide | Array | Location | Antibody | Type |
| --- | --- | --- | --- | --- |
| 1 | 1 |  | RABBIT CONTROL |  |
|  | 2 | 3 | ATM/ATR Substrate P Ser/Thr | rabbit |
|  | 3 | 4 | Aurora A/B/C P Thr288/Thr232/Thr198 | rabbit |
|  | 4 | 5 | Bad P Ser136 | rabbit |
|  | 5 | 6 | Bad P Ser112 | rabbit |
|  | 6 | 7 | Bak | rabbit |
|  | 7 | 12 | IkB-alpha | rabbit |
|  | 8 | 19 | PARP | rabbit |
|  | 9 | 21 | PI3 Kinase p110-alpha | rabbit |
|  | 10 | 23 | PKC (pan) P Ser660 (beta-2) | rabbit |
|  | 11 | 24 | PKC substrate P (R/K)X(S*)(Hyd)(R/k) | rabbit |
|  | 12 | 25 | PKC-zeta | rabbit |
|  | 13 | 26 | PKC-zeta/lambda P Thr410/403 | rabbit |
|  | 14 | 27 | Bim | rabbit |
|  | 15 | 42 | IkB-alpha P Ser32 | rabbit |
|  | 16 | 43 | S6 Ribosomal protein P Ser235,Ser236 | rabbit |
| 2 | 1 |  | RABBIT CONTROL |  |
|  | 2 | 49 | PARP cleaved Asp214 | rabbit |
|  | 3 | 51 | NFkB p65 Ser536 | rabbit |
|  | 4 | 52 | cdc25c P Ser216 | rabbit |
|  | 5 | 53 | Chk1 P Ser345 | rabbit |
|  | 6 | 54 | Chk2 P Thr68 | rabbit |
|  | 7 | 58 | Rb | rabbit |
|  | 8 | 65 | cdc25A | rabbit |
|  | 9 | 67 | PTEN | rabbit |
|  | 10 | 70 | Tsc-2 (Tuberin) P Thr1462 | rabbit |
|  | 11 | 71 | Tsc-2 (Tuberin) | rabbit |
|  | 12 | 72 | Survivin | rabbit |
|  | 13 | 73 | p70 S6 Kinase P Thr389 | rabbit |
|  | 14 | 75 | Cyclin D1 | rabbit |
|  | 15 | 76 | SAPK/JNK (JNK2) | rabbit |
|  | 16 | 77 | p70 S6 Kinase P Thr421,Ser424 | rabbit |
| 3 | 1 | 81 | p53 P Ser15 | rabbit |
|  | 2 | 90 | PDK-1 P Ser241 | rabbit |
|  | 3 | 93 | p70 S6 Kinase | rabbit |
|  | 4 | 97 | S6 Ribosomal protein p Ser240,Ser244 | rabbit |
|  | 5 | 98 | S6 Ribosomal Protein | rabbit |
|  | 6 | 99 | Rb P Ser807,Ser811 | rabbit |
|  | 7 | 100 | Rb P Ser780 | rabbit |
|  | 8 | 103 | Bax | rabbit |
|  | 9 | 107 | Smad2/3 P Ser465/Ser423,Ser467/Ser425 | rabbit |
|  | 10 | 108 | Smad1/5 P Ser463/Ser465 | rabbit |
|  | 11 | 109 | Cyclin D1 P Thr286 | rabbit |
|  | 12 | 112 | Bcl-2 | rabbit |
|  | 13 | 113 | Bcl-xl | rabbit |
|  | 14 | 114 | Bid | rabbit |
|  | 15 | 115 | Bim P Ser69 | rabbit |
|  | 16 | 117 | Caspase 3 | rabbit |
| 4 | 1 | 118 | Caspase 3 cleaved | rabbit |
|  | 2 | 119 | CDK1 (p34cdc2) P Tyr15 | rabbit |
|  | 3 | 120 | p53 | rabbit |
|  | 4 | 121 | CREB | rabbit |
|  | 5 | 137 | SQSTM1 (p62) | rabbit |
|  | 6 | 139 | Puma | rabbit |
|  | 7 | 141 | Smad2 P Ser465,Ser467 | rabbit |
|  | 8 | 143 | VEGFR P Tyr951 | rabbit |
|  | 9 | 146 | CDK2 | rabbit |
|  | 10 | 149 | Tau Phospho/non Phos ser 305 | rabbit |
|  | 11 | 151 | XIAP | rabbit |
|  | 12 | 154 | NFkB p105/p50 | rabbit |
|  | 13 | 160 | Bcl-2 P Ser70 | rabbit |
|  | 14 | 162 | Gab1 | rabbit |
|  | 15 | 168 | Rock1 (C8F7) | rabbit |
|  | 16 | 172 | Arf6 (D12G6) | rabbit |
| 5 | 1 | 204 | Histone H2A.X P Ser139 [EP854(2)Y] | rabbit |
|  | 2 | 208 | EphB3 [EPR8280] | rabbit |
|  | 3 | 214 | Lamin A/C | rabbit |
|  | 4 | 218 | Ku80 (C48E7) | rabbit |
|  | 5 | 222 | FOXO3a (75D8) | rabbit |
|  | 6 | 225 | FOXO1 (C29H4) | rabbit |
|  | 7 | 227 | Smad2 (C86F7) | rabbit |
|  | 8 | 228 | Ezrin P T567/Radixin T564/Moesin T588 | rabbit |
|  | 9 | 251 | Smad1 (D59D7) | rabbit |
|  | 10 | 260 | c-Abl P Y245 | rabbit |
|  | 11 | 261 | c-Abl P Y412 (247C7) | rabbit |
|  | 12 | F03 | ATM | rabbit |
|  | 13 |  | Fox01 P | rabbit |
|  | 14 |  |  |  |
|  | 15 |  |  |  |
|  | 16 |  |  |  |
| 6 | 1 |  | MOUSE CONTROL |  |
|  | 2 |  | MOUSE CONTROL |  |
|  | 3 | 28 | CDK1 (cdc2) | mouse |
|  | 4 | 87 | p21 CIP/WAF1 | mouseIgG2a |
|  | 5 | 130 | PKC-alpha | mouseIgG2b |
|  | 6 | 155 | Calmodulin | mouseIgG |

**Supplementary Table S4.** List of antibodies used for reverse phase protein array (RPPA) analysis, including phosphosite-specific epitopes where applicable.

| Antibody target | Antibody category | Phosphosite / epitope |
| --- | --- | --- |
| ATM/ATR substrate | Phospho-specific | Ser/Thr |
| Aurora A/B/C | Phospho-specific | Thr288/Thr232/Thr198 |
| BAD | Phospho-specific | Ser136 |
| BAD | Phospho-specific | Ser112 |
| BAK | Total protein | — |
| IκBα | Total protein | — |
| IκBα | Phospho-specific | Ser32 |
| PARP | Total protein | — |
| PARP (cleaved) | Cleavage-specific | Asp214 |
| p53 | Total protein | — |
| p53 | Phospho-specific | Ser15 |
| FOXO1 | Total protein | — |
| FOXO1 | Phospho-specific | unspecified |
| Caspase-3 | Cleaved form | cleaved |
| CDK1 (cdc2) | Total protein | — |
| CDK1 (p34cdc2) | Phospho-specific | Tyr15 |
